# An Exploration of Domain-Specific Sedentary Behaviors in College Students by Lifestyle Factors and Sociodemographics

**DOI:** 10.3390/ijerph18189930

**Published:** 2021-09-21

**Authors:** Chelsea Carpenter, Sang-Eun Byun, Gabrielle Turner-McGrievy, Delia West

**Affiliations:** 1 Department of Clinical and Health Psychology, College of Public Health and Health Professions, University of Florida, Gainesville, FL 32610, USA; clarsen1@phhp.ufl.edu; 2 Department of Retailing, College of Hospitality, Retail and Sport Management, University of South Carolina, Columbia, SC 29208, USA; sbyun@mailbox.sc.edu; 3 Department of Health Promotion, Education and Behavior, Arnold School of Public Health, University of South Carolina, Columbia, SC 29208, USA; brie@sc.edu; 4 Department of Exercise Science, Arnold School of Public Health, University of South Carolina, Columbia, SC 29208, USA

**Keywords:** screen-time, sedentary behavior, adolescents, obesity

## Abstract

College students exhibit high levels of sedentary time and/or poor lifestyle factors (e.g., poor sleep, stress, physical inactivity). It is unknown; however, in what domains college students spend their sedentary time and whether there are associations between sedentary time and these lifestyle factors. This study examined sedentary behavior of college students by domains, current lifestyle factors and sociodemographics. Undergraduates (n = 272, M age = 20 years, 79% female) self-reported their sedentary behavior, sleep, stress, physical activity, anthropometrics and sociodemographics. Sedentary time was categorized as: total, recreational screen, education and social. Students reported spending > 12 h of their day sedentary on average, with over a third of this time spent in recreational screen time. All categories of sedentary time were significantly correlated with body mass index, and both total sedentary time and screen time were significantly correlated with sleep score, with poorer sleep quality associated with greater sedentary time. Physical activity was negatively correlated with social sedentary time only. Subgroups with elevated sedentary time included minority students, those with low parental education and students with overweight/obesity. Given the negative health impacts of sedentary behavior, college students would likely benefit from interventions tailored to this population which target reducing sedentary time, particularly recreational screen time.

## 1. Introduction

Emerging epidemiologic evidence suggests an independent and positive association between high levels of sedentary time and increased risk of chronic conditions, such as cardiovascular disease [1], hypertension [2], and type 2 diabetes [3,4]. When examined by age, adolescents and young adults are one of the most sedentary subgroups, with estimates of over 8 h of sedentary time per day [5]. Despite the high levels of sedentary behavior among young adults, there has been little research examining characteristics of sedentary time within this population.

Insights into the sedentary behavior of college students are of particular interest given the sizeable proportion of young adults who attend college [6] and the nature of the undergraduate experience which incorporates so many sedentary activities. Accelerometery data estimate that college students spend nearly 10 h per day engaged in sedentary behavior [7], with a majority of that time spent sitting in class or studying [8]. This is particularly worrisome given evidence that lifestyle habits developed during the transition from adolescence into adulthood track well into later life [9]; therefore, sedentary college students are likely to become sedentary adults, and experience all the negative health sequalae associated with sedentary patterns [10,11].

Despite the apparent high levels of sedentary behavior among undergraduate students and the risks associated with sedentary patterns, few studies have characterized the domains in which college students spend their sedentary time and identified subgroups of undergraduates at highest risk of elevated sedentary time. For example, there is evidence of racial differences in sedentary behavior in adults [12,13,14,15] and suggestions that screen time and homework time may vary by sex [16], but a more robust exploration of the full range of sedentary behavior domains across subgroups of college students would aid in parsing out those individuals to whom sedentary behavior reduction interventions might be targeted.

Furthermore, there is growing interest in how sleep quality, physical activity and mood are associated with sedentary time across the relevant domains. Research in other populations indicates that screen time is the most common sedentary activity [17], and screen time has been associated with poor sleep quality [18,19,20], decreased physical activity [19,21], and higher body weight among adolescents [22,23,24,25], as well as poor sleep quality [26,27] and higher body mass index (BMI) [28,29] among adults. There are initial indications that stress [30], poor sleep [31] and low levels of physical activity [32] might be associated with sedentary behavior among college students. However, no studies of which we are aware have examined the range of sedentary behavior domains among college students relative to these other important lifestyle factors.

Thus, the purpose of this study was to characterize the extent and domain-specific patterns of sedentary behavior among college students, to explore sedentary profiles across sociodemographic subgroups, and to examine relationships between sedentary patterns and other lifestyle factors. We hypothesized that total sedentary time would be higher among minority students, individuals with overweight or obesity, and individuals with a lower physical activity level. In addition, we hypothesized that screen time would be associated with poorer sleep quality, weight status, and lower levels of physical activity.

## 2. Materials and Methods

### 2.1. Study Design

This cross-sectional study assessed self-reported sedentary behavior, stress, sleep and physical activity among undergraduate students at a major public southeastern university.

### 2.2. Participants

Current undergraduate students were eligible to participate without any other inclusion criteria. Participants were recruited in the fall semester of 2018 through flyers, listservs, and announcements made by course instructors in various colleges. Interested students were directed to a study website, where they completed the online informed consent and an online survey via a unique and secure link (REDCap, Vanderbilt, TN, USA). The 272 students who completed the survey were entered into a gift card drawing. All participants gave their informed consent before inclusion in the study, which was approved by the University of South Carolina Institutional Review Board.

### 2.3. Measures

*Sedentary Behavior.* The Sedentary Behavior Questionnaire (SBQ) is an 8-item self-report measure of time spent engaged in sedentary activities on weekdays and weekend days separately across a range of domains. It has been shown to be reliable and valid [33] and was adapted to reflect current technologies likely to be most relevant to college students (e.g., replacing videocassette recorder (VCR) with smartphone or tablet), to expand descriptions to include activities common to college students (e.g., attending class, doing coursework, school-related computer time) and to add an item about sedentary socializing (i.e., at coffeeshop, sports event, bar or house). Mean daily sedentary time was calculated using a weighted average of weekday and weekend day sedentary time [34]. In addition to quantifying total sedentary time, domain-specific sedentary time was calculated for leisure screen time, educational/work time, and socializing (on phone and in person). Any total sedentary time values greater than 1440 min (i.e., 24 h), were truncated to 1440 min [33,34].

*Physical Activity.* Physical activity was assessed using the International Physical Activity Questionnaire (IPAQ) short form, which has been shown to be valid and reliable for young adults [35,36]. The IPAQ quantifies physical activity accrued in bouts of at least ten minutes over the previous week, measuring frequency and duration of vigorous and moderate intensity physical activity, walking, and sitting time. This allows the calculation of metabolic equivalent (MET) minutes per week and gives an indication of energy expenditure. It also allows the classification of individuals as low active, moderately active, or high active using established metrics [37]. However, because there were few low active individuals in the sample of this study, low and moderately active students were combined into a single group for analysis purposes.

*Sleep* was assessed using the valid and reliable Pittsburgh Sleep Quality Index (PSQI) [38]. The PSQI is a 19-item questionnaire that evaluates seven components of sleep, including sleep duration, sleep disturbance, sleep latency, daytime dysfunction due to sleepiness, sleep efficiency, overall sleep quality and sleep medication use. The Global PSQI score (range 0–21) allows classification into individuals with poor sleep quality (score > 5) and good sleep quality (score ≤ 5). The measure has been used in studies which examine relationships between sleep and physical activity among college students [39].

*Stress* was assessed using the 10-item Perceived Stress Scale (PSS) [40] to determine the degree to which individuals perceived their lives as stressful over the previous month. Scores range from 0 to 40 and are categorized into low (0–13), moderate (14–26), or high (27–40) stress [40].

*Demographic variables* assessed included age, sex, race/ethnicity, and whether their parent(s) went to college. Height and body weight were self-reported and body mass index was then calculated (kg/m^2^) and categorized according to CDC guidelines into underweight, normal weight, overweight and obese [41]. Students with overweight or obesity were grouped together and compared with underweight and normal weight students.

### 2.4. Statistical Analyses

Descriptive statistics for continuous variables were calculated using means and standard deviations. Categorical variables were described using frequencies and percentages. Independent *t*-tests were used to examine whether sedentary behavior differed by sex, race/ethnicity, parental college attendance, weight category, physical activity level category or sleep category. One-way ANOVA was conducted to examine whether there were differences in sedentary behavior across the stress categories. Pearson’s correlation was used to examine whether there were correlations between sedentary behavior domains and weight, physical activity, sleep and stress variables. Correlation strength was defined as small (0.1–0.3), medium (0.3–0.5), and large (0.5–1.0) [42]. All analyses were performed using SAS version 9.4 (SAS Institute, Cary, NC, USA).

## 3. Results

A total of 354 young adults consented to participate in the study. A total of eighty-two individuals were excluded from analyses because they did not complete the survey (*n* = 78), were enrolled in a graduate program (*n* = 1) or were not currently enrolled at the university (*n* = 3). This left a sample of 272 eligible undergraduates. Overall, participants were predominately white females (79%) of normal weight (71%), reporting high levels of physical activity (2514 MET minutes/week), moderate perceived stress and poor sleep quality. Sample characteristics can be found in Table 1.

### 3.1. Sedentary Behavior Time across Domains

On average, undergraduates reported spending a total of 12.7 ± 5.6 h per day engaged in sedentary behavior. The largest proportion of their sedentary time was spent engaged in recreational screen-related sedentary activities, accounting for approximately 35% of sedentary time at 4.5 ± 2.8 h. Sedentary time spent in educational activities and in socializing accounted for 27% and 24% of sedentary behavior, respectively, with an average of 3.4 ± 2.2 h spent in school and work-related sedentary activities and 3.0 ± 2.1 h spent sedentary while socializing.

Overall and domain-specific sedentary behavior patterns differed by demographic characteristics (see Table 2). Although female students reported similar amounts of total time spent sedentary to males, and comparable time in sedentary educational pursuits and recreational screen time, female undergraduates reported significantly more sedentary time socializing than males. Minority students reported significantly greater overall sedentary time than white students. Furthermore, minority undergraduates reported more time engaged in sedentary educational activities and significantly greater recreational screen time than did white college students. Indeed, the only domain in which there were no differences between minority and white students in sedentary time was sedentary socializing.

Among students reporting lower parental education, total sedentary time was significantly greater than was reported by students with higher parental education. Students with ≤1 parent who attended college also spent significantly more time engaged in sedentary educational/work-related activities than did students who had two parents that attended college. However, leisure screen time and sedentary socializing were similar between the two groups.

### 3.2. Sedentary Time and Other Health Variables

Weight category (e.g., underweight/normal weight vs. overweight/obese) was significantly associated with sedentary time among undergraduates, with students with overweight or obesity reporting significantly greater total sedentary time than students who were normal weight or underweight. Those who were classified as overweight or obese also reported significantly greater time in sedentary socializing. However, there was no difference between students of differing weight groups with respect to educational sedentary time or screen time. Similarly, students who were classified as low/moderate active had significantly greater total sedentary time and significantly greater social sedentary time compared with those who were high active but did not differ in screen time or educational sedentary time.

Total sedentary time and screen time were both significantly higher among individuals with poor sleep quality compared to those with good sleep quality. However, no differences in sedentary time were apparent among students with differing levels of stress.

**Figure 1:**
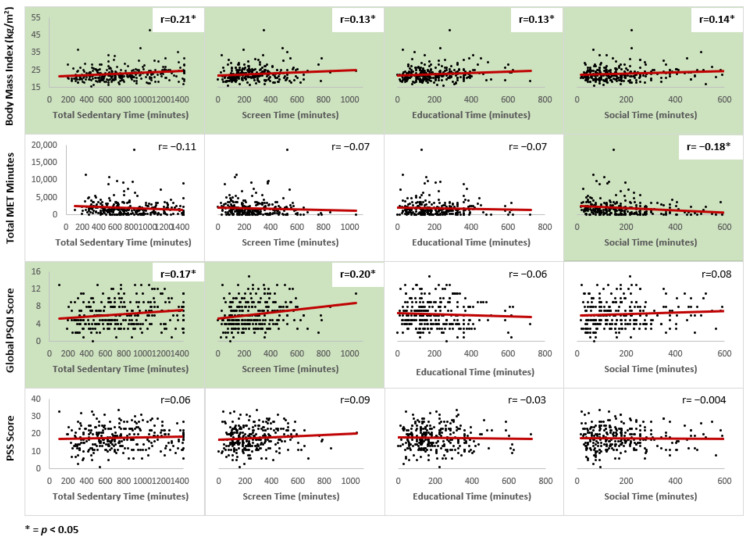
Correlations between sedentary behavior domains and lifestyle factors.

To explore the strength of associations between sedentary behavior and these other lifestyle factors, we examined the correlations between total sedentary time, domain-specific sedentary time and the lifestyle factors of interest (Figure 1). There were significant but small positive correlations between BMI and overall sedentary time, screen time, educational sedentary time and socializing time. Total MET minutes had a small but significant negative correlation with social sedentary time, but not any of the other sedentary behavior domains. Global PSQI sleep score had small significant positive associations with both total sedentary time and screen time. There were no significant associations between stress level and any of the sedentary behavior parameters.

## 4. Discussion

College students engaged in substantial amounts of sedentary time, reporting an average sitting time of over 12 h a day. The majority of sedentary time was accrued in recreational screen time, with students reporting 4.5 h of daily leisure screen time. This is double the 1.5–2.4 h per day of screen time reported in older studies of college populations [16,29,43]. Differences between these previous studies and the current findings may reflect how screen time was operationalized; some previous studies included only TV viewing as screen time [43] and others combined multiple screen-based activities into screen time [16,29]. The comprehensive range of screen formats noted in survey items for the current study may have served as a prompt to remind students of the spectrum of activities to consider when responding about screen time, and that might have contributed to the higher report of sedentary time. The current results, however, are comparable to a recent study which reported 4 h of screen time among college students in Spain. Importantly, these data were confirmed by objective measurement [30]. Moving forward, to fully capture the time young adults spend engaged in recreational screen-related activities, it will be critical to include a comprehensive list of all possible screens; the evolution of technology has created the ability to access content on multiple types of screens and not just TVs. Thus, asking only about time spent viewing TV runs the risk of missing a considerable amount of screen time.

Education and work-related sedentary time accounted for 3.4 h of daily sedentary time, which is more than double the homework time reported a previous study of undergraduates [16]. This too might reflect how educational sedentary time was operationalized in the current study. We included not only doing homework, but also time sitting in class and doing course-related work on the computer to capture the full range of sedentary educational pursuits. There are emerging data that it is feasible to reduce sedentary time in the college classroom setting [44,45,46] through the implementation of environmental changes, such as installing standing desks or encouraging standing in lectures, which may help reduce educational sedentary behavior without decreasing the time spent in educational pursuits.

This is one of the first studies we are aware of to have examined sedentary time spent socializing. Undergraduates reported engaging in 3 h a day of sedentary socializing, indicating that this domain merits greater attention. Our results showed that female undergraduates spent more time engaged in sedentary socializing than males. Others have observed that college females are more likely to spend time using social media than males [47], but the current study expands socializing to include both media-based and in-person socializing. Other subgroups that indicated greater sedentary socializing in the current study included those who were overweight or obese and those who engaged in low levels of physical activity, which are subgroups that likely overlap. Since socializing is a significant part of the lifestyle of college students, and is often accomplished while sedentary, it may represent a “hidden” aspect of undergraduate sedentary time which requires greater quantification and characterization to determine whether it is amenable to modification to reduce overall sedentary time.

Minority students were particularly likely to spend substantial amounts of time engaged in sedentary behaviors, with 6 h a day engaged in recreational screen time, which is almost 2 h more than the screen time reported by white students. This was accompanied by higher amounts of sedentary time spent engaged in educational activities as well, resulting in significantly higher overall sedentary time among minority undergraduate students. Although this is not the first study to report greater screen time among minorities [48], it is the first of which we are aware to highlight racial differences in education-based sedentary time. These data echo studies noting racial differences among adults in sedentary behavior [12,13,14,15], and suggest that sedentary habits develop early in young adulthood and track into later life. Interventions to reduce sedentary behavior designed to reach minority undergraduate students could interrupt this pattern and reduce known health disparities in conditions related to sedentary behavior, such as cardiovascular disease and type 2 diabetes [1].

First-generation college students and those with lower parental education were another group that emerged from these analyses as a likely sedentary population and thus worthy of consideration for health promotion efforts. Students with lower parental education had greater overall sedentary time than students with higher parental education, as well as greater sedentary time spent engaged in educational/work activities. This is consistent with studies examining the role of parental education on screen time that report an association between lower parental educational attainment and higher screen time [18,49]. However, this is the first study we are aware of that examined educational sedentary time among students with low parental education. Undergraduates with lower parental education are less likely to graduate and to have other indicators of academic distress [50,51,52]. The current findings that they spend a greater amount of time engaged in educational and work-related sedentary behaviors may signal that they are either struggling with their coursework and thus spending more time sedentary while doing this coursework or that they spend more time working at sedentary jobs than do students with higher parental education. Since young adults with lower parental education tend to have a worse health profile than do students from families with higher parental education [53], sedentary behavior reduction may offer a novel lifestyle behavior to target to ameliorate some of that elevated risk.

Sleep quality, but not perceived stress, was associated with elevated sedentary behavior. Of particular note is the relationship between elevated recreational screen time and poor sleep quality seen in this study. Associations between poor sleep and both objectively measured sedentary time [31] and screen time [19,29] have previously been reported among college students. In this cross-sectional study, it cannot be ascertained whether increased screen time resulted in poor sleep or vice versa. If excessive screen time were a determinant of poor sleep, reducing screen time may be an important and attractive behavioral intervention target for undergraduates given the association between poor sleep and decreased academic performance [54,55] and impaired mental health [55].

Although this study advances our understanding of sedentary patterns among undergraduate students and points to specific groups at risk of greater time spent sedentary, the study is not without limitations. Foremost is the reliance on a self-reported measure of sedentary behavior. Self-report offers the advantage of elucidating domains of sedentary behavior, but self-report can underestimate time spent sedentary [56,57], particularly among college students [58]. Objective measurement coupled with self-report is considered the optimal approach to ascertaining sedentary patterns [12,59]. Furthermore, there exists the possibility that students over-reported their time engaged in sedentary behaviors, as the current generation of undergraduates tends to multi-task with media [60], and thus, time spent in sedentary activities could be counted twice across multiple domains (e.g., texting while watching TV) [61]. However, these considerations would impact the precision of the measurement rather than the comparative patterns which emerged, suggesting that results highlighting particular subgroups at risk for elevated sedentary time who might benefit from targeted sedentary reduction interventions to mitigate associated negative health consequences are likely robust. Other study limitations are the cross-sectional design and lack of sufficient sample size within individual minority subgroup populations to explore comparisons within specific racial-ethnic groups to pinpoint those subgroups at highest risk for a deleterious pattern of sedentary behavior. A larger sample would also enable the examination of low and moderate active students separately, as well as the full range of weight categories. Future studies could also build upon the *t*-tests used in this study and employ multivariate analyses to examine differences within each of the identified subgroups, which would provide informative next steps for this research. Finally, whether physical activity negates sedentary behavior’s effects on health [62] (or vice versa) and how sedentary behavior should be addressed within the broader context of health promotion are both areas for continued discussion.

## 5. Conclusions

College students engage in a substantial amount of sedentary behavior, with sedentary time accrued across several important domains, including recreational screen time, educational activities, and socializing. Subgroups that are at particular risk for elevated sedentary time (and therefore higher risk of the negative health consequences associated with sedentary time) include minority students, those with low parental education and students with overweight/obesity. Targeted interventions to reduce sedentary time might be considered for these subgroups. However, it is important to note that although sedentary behavior was elevated in these subgroups, it was also quite high in their counterparts. In all pairs of subgroups compared, even the “healthier” of the two groups engaged in at least 12 h of sedentary behavior each day. Therefore, all undergraduates would likely benefit from sedentary behavior reductions. Designing interventions that meet the unique needs of undergraduates and address sedentary behavior across the range of domains would likely be most effective.

## Figures and Tables

**Table 1:** Sample (*n* = 272) characteristics.

Demographic and Lifestyle Factors	
Sex	
Female	216 (79%)
Male	56 (21%)
Age (M ± SD)	20.0 ± 1.5 years
Mean Body Mass Index (M ± SD)	22.9 ± 3.8 kg/m^2^
Body Mass Index Category, *n* (%)	
Under weight (BMI < 19)	17 (6%)
Normal weight (19 ≤ BMI > 25)	194 (71%)
Overweight (25 ≤ BMI > 30)	48 (18%)
Obese (BMI ≥30)	13 (5%)
Race, *n* (%)	
White	232 (85%)
Minority ^a^	40 (15%)
Ethnicity, *n* (%)	
Not Hispanic	263 (97%)
Hispanic	9 (3%)
College, *n* (%)	
Arts & Sciences	23 (9%)
Business	5 (2%)
Hospitality, Retail and Sport Management	101 (37%)
Nursing	21 (10%)
Public Health	115 (42%)
Class Standing, *n* (%)	
Freshman	31 (12%)
Sophomore	71 (26%)
Junior	88 (32%)
Senior	82 (30%)
Number of Parents who Attended College, *n* (%)	
Zero	34 (13%)
One	72 (26%)
Two	166 (61%)
Pittsburgh Sleep Quality Inventory Score (M ± SD)	6.2 ± 2.8
Perceived Stress Scale Score (M ± SD)	17.8 ± 6.2
International Physical Activity Questionnaire derived MET Minutes/Week (Interquartile Range [IQR])	
Low/Moderate Levels of Physical Activity	547.7 [817]
High Levels of Physical Activity	2513.7 [1901]

^a^: Black/African American, Native American, Asian & multiracial.

**Table 2:** Domain-specific sedentary behavior by sociodemographic and lifestyle factors.

	Hours of Self-Reported Daily Sedentary Time (Mean ± SD)			
	Total	Screen Time	Education	Social
Sex				
Male (*n* = 56) Female (*n* = 216)	12.5 ± 6.0	4.2 ± 2.4	3.1 ± 2.0	2.3 ± 1.5 *
	12.7 ± 5.5	4.5 ± 2.9	3.5 ± 2.2	**3.2 ± 2.2**
Race				
White (*n* = 232) Minority (*n* = 40)	12.1 ± 5.3 *	4.2 ± 2.5 *	3.3 ± 2.1 *	3.0 ± 2.1
	**15.7 ± 6.5**	**6.1 ± 3.8**	**4.1 ± 2.5**	3.1 ± 2.3
Parent College				
Neither or one attended (*n* = 106) Both attended (*n* = 166)	13.6 ± 5.9 *	4.9 ± 3.1	3.8 ± 2.1 *	3.1 ± 2.4
	**12.1 ± 5.4**	4.2 ± 2.5	**3.2 ± 2.2**	2.9 ± 1.9
Weight Category (BMI)				
Underweight/Normal weight (*n* = 211) Overweight/Obese (*n* = 61)	12.1 ± 5.4 *	4.3 ± 2.8	3.3 ± 2.2	2.9 ± 2.0 *
	**14.6 ± 6.0**	5.0 ± 2.8	3.8 ± 2.3	**3.5 ± 2.4**
Physical Activity Category (IPAQ)				
Low/Moderate (*n* = 126) High (*n* = 146)	13.5 ± 5.4 *	4.8 ± 2.9	3.3 ± 2.0	3.4 ± 2.2 *
	**12.0 ± 5.7**	4.2 ± 2.7	3.4 ± 2.3	**2.6 ± 2.0**
Sleep Category (PSQI)				
Poor Sleep (*n* = 146) Good Sleep (*n* = 126)	13.6 ± 5.6 *	5.0 ± 2.9 *	3.3 ± 2.1	3.2 ± 2.2
	**11.6 ± 5.5**	**3.9 ± 2.6**	3.5 ± 2.3	2.7 ± 1.9
Stress Category (PSS)				
Low (*n* = 74) Medium (*n* = 172) High (*n* = 26)	13.2 ± 5.2	4.1 ± 2.7	3.7 ± 2.4	2.9 ± 2.3
	12.1 ± 5.9	4.6 ± 2.9	3.2 ± 2.1	3.0 ± 2.0
	12.8 ± 5.6	4.6 ± 2.6	3.6 ± 2.1	3.1 ± 2.1

Bolded numbers and *: statistically significant differences between groups, *p* < 0.05.

## Data Availability

Research data are not available because participant consent did not include sharing of data.
